# The Control of Measles and Whooping-Cough

**Published:** 1898-05-07

**Authors:** 


### THE CONTROL OF MEASLES AND WHOOPING-COUGH.

An interesting question has been raised in Scotland in regard to the interpretation of Section 57 of the Public Health (Scotland) Act, and it is all the more interesting to us from the fact that its answer would seem to apply equally to that section (126) of the Public Health Act, 1875, which renders it unlawful for any person suffering from a dangerous infectious disorder to wilfully expose himself, without proper precautions against spreading the disorder, in any street, public place, shop, inn, &c. People often speak as if the sanitary authorities were helpless in regard to the control of those infectious diseases, such as measles and whooping-cough, which are not scheduled under the Notification Act, and, in fact, the schedule of this latter Act is commonly looked upon as defining what is meant by a dangerous infectious disorder. But this is an error. The two Acts have no special connection the one with the other.

The question raised in Scotland had to do with a clause which imposes a penalty upon parents and school teachers who knowingly permit a child to attend school who has been suffering from infectious disease, or who has been residing in a school where such disease exists, unless the child in question produces a medical certificate stating that he or she is free from infection. In view of this clause, the Scotch Local Government Board were asked whether measles and whooping-cough were to be held to be infectious diseases, even although a sanitary authority might not have added these two diseases to those notifiable under the Notification Act. In answer to this inquiry, the Board have laid it down that the provisions of the Public Health Act are in no wise limited to the diseases mentioned in the Notification Act, and that both measles and whooping-cough are dangerous infectious diseases, and must be dealt with as the Act requires.

We see no reason why English sanitary authorities should not act on the same rule in regard to the exposure in public places not only of patients suffering from these diseases, but of clothing, or other things, which have been exposed to the infection, and especially in regard to the letting of lodgings (Section 127), and the making of false answers in regard to cases of such diseases, having occurred in such lodgings (Section 129). In any epidemic the sanitary officers can always find out the addresses of the infected houses from the school authorities, and in that way may put themselves in almost as good a position in regard to the control of these diseases as if they were notifiable.

